# Functional genomics in *Trypanosoma brucei*: A collection of vectors for the expression of tagged proteins from endogenous and ectopic gene loci

**DOI:** 10.1016/j.molbiopara.2007.03.012

**Published:** 2007-07

**Authors:** Steven Kelly, Jenny Reed, Susanne Kramer, Louise Ellis, Helena Webb, Jack Sunter, Jeanne Salje, Nina Marinsek, Keith Gull, Bill Wickstead, Mark Carrington

**Affiliations:** a Sir William Dunn School of Pathology, University of Oxford, South Parks Road, Oxford OX1 3RE, UK; b Department of Biochemistry, University of Cambridge, 80 Tennis Court Road, Cambridge CB2 1GA, UK

**Keywords:** *Trypanosoma brucei*, Functional genomics, Vectors, Transgene expression

The expression of transgenes encoding proteins modified to contain residues that impart a particular property, ‘tagged proteins’, is central to the post-genomic analysis of any organism. *Trypanosoma brucei* is a model kinetoplastid protozoan pathogen and has the most advanced repertoire of tools for reverse genetic analysis available for any protozoan. The vast majority of these tools take advantage of the predominance of homologous over non-homologous recombination to target constructs to specific genomic loci. Initially the targeting was used to direct unregulated transgenes to transcribed regions of the genome [1] and to perform gene deletions [2,3]. A leap forward in the sophistication of reverse genetic experiments occurred with the development of trypanosome cell lines expressing the tetracycline repressor (TetR) protein which facilitated tetracycline-regulated expression of transgenes [4,5]. Further development of cell lines expressing both TetR and bacteriophage T7 RNA polymerase (T7RNAP) allowed transgenes to be transcribed and expressed at very high levels [6]. The TetR- and T7RNAP-expressing cell lines are also central to most RNA interference-based analyses of gene function currently performed in trypanosomes [7–10]. In nearly all cases, an antibiotic resistance gene is used as the selectable marker. The DNA used for targetted integration is usually a linearised plasmid; targetting using PCR products directly is possible [11–13] but integration is less efficient and does not usually offer inducible expression.

The expression of tagged proteins has become central to the technologies that have developed to analyse the function of individual genes. The tag can have a range of functions falling into two main categories: the first is to provide evidence for the sub-cellular localisation of a protein in living cells using a fluorescent protein tag (see, for example [14]) or in fixed cells using a fluorescent protein or epitope tag (see, for example [15]). The second is to facilitate the purification of complexes which, when allied with mass spectrometric analysis and knowledge of the genome sequence, allows the identification of components of multimeric proteins. Two types of tag have been developed successfully in yeast: (i) the tandem affinity tag where two successive rounds of purification are used [16], and (ii) a tandem epitope tag which is used in a single step purification [17]. To date, the former has been exploited more in trypanosomes [18,19]. In the future, the investigation of transient protein-protein interactions *in vivo* could be analysed by techniques such as fluorescence resonant energy transfer (FRET) which is dependent on tagging the two target proteins with different fluorescent proteins [20]. Here, we describe five sets of plasmids that represent a substantial collection of publicly available vectors for adding tags to the N- and C-termini of proteins in *T. brucei*.

## 1 New vectors for inducible expression of transgenes from ectopic loci

Vectors were based on three different backbones: pLEW100 [6] (kind gift of George Cross, Rockerfeller University), and two new vectors, pDex377 and pDex577. Transgene expression in pLEW100 is regulated by a tetracycline-inducible EP1 procyclin promoter [6]. The plasmid was designed to integrate into the non-transcribed spacer between ribosomal RNA (rRNA) genes and requires cell lines expressing T7RNAP, as the bleomycin resistance selectable marker (*bleR*) is transcribed by a modified T7RNAP promoter. pDex377 is a new plasmid derived from pLEW100 and uses the same tetracycline-inducible EP1 procyclin promoter for control of transgene expression [6]. However, in pDex377 the selectable marker was changed to a hygromycin resistance gene (*hygR*) under the control of a rRNA promoter and the targeting sequence was changed to a repetitive DNA present on minichromosomes and intermediate-sized chromosomes (the 177 bp repeat). Regulated expression of the transgene from pDex377 requires cell lines expressing the tetracycline repressor only, but the vector can be used for unregulated expression in any cell line. pDex577 is a new plasmid that was designed to produce high level over-expression of proteins. It was derived in part from p2T7-177 [21] and pLEW100; transgene expression is directed by a tetracycline-inducible T7 promoter. pDex577 contains a *bleR* gene transcribed by a rRNA promoter and is targeted to 177 bp repeats [21]. The new plasmids pDex377 and pDex577 were sequenced to completion (4× coverage) and all additional tags/modifications were verified by sequencing. The plasmids are available from the authors and the sequences of all vectors are available from http://web.mac.com/mc115/iWeb/mclab/home.html and http://users.path.ox.ac.uk/∼kgull/index.html.

**Fig. 1:**
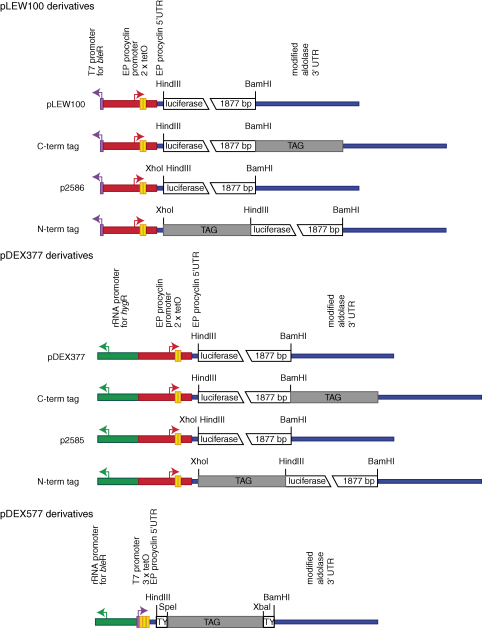
Diagram showing the structure of the tetracycline-regulated transgene expression vectors described herein. The *Hin*dIII and *Bam*HI cloning sites are indicated. Red, EP1 procyclin promoter; yellow, tet operators; lilac, T7 promoter; blue, transgene 5′ and 3′UTR; green, ribosomal RNA promoter; TY, TY epitope tag. The plasmids p2586 and p2585 were intermediates in the production of the N-terminal tagging vectors. To produce the C-terminal tagging vectors, pLEW100 was digested with *Bam*HI and the tags inserted as *Bam*HI *Bgl*II fragments. For the N-terminal tagging vectors, pLEW100 was digested with *Hin*dIII and a synthetic polylinker containing an *Xho*I site inserted to produce p2586. The resultant vector allowed directional insertion of tags as *Xho*I *Hin*dIII fragments. The identity and veracity of all inserts was confirmed by sequencing. pDex377 was derived from pLEW100 by replacement of the targeting sequence and the selectable marker including its promoter. DNA containing the 177 bp repeat targeting sequence was removed from p2T7-177 as a *Pvu*I–*Kpn*I fragment (blunted at the *Kpn*I site with Klenow) and cloned into *Pvu*I *Sac*II (blunt) digested pLEW100. The resulting construct, pDex177, was digested with *Kpn*I, blunted with Klenow, then digested with *Sal*I and then ligated to a *Sal*I–*Sph*I (blunt) fragment containing the rRNA promoter and *HygR* gene from p2T7tiTA (kind gift of David Horn, London School of Hygiene and Tropical Medicine, UK). The sequence of pDex377 was confirmed by sequencing the whole plasmid to 4× coverage. The pDex377-derivatives were made in the same way as the pLEW100 derivatives (see above), p2585 was the intermediate plasmid for N-terminal tagging vectors. pDex577 was derived from p2T7-177 and pLEW100. The luciferase gene, encompassing 5′ and 3′UTRs and two tetracycline operators were amplified from pLEW100 with *Kpn*I and *Xho*I sites incorporated into the 5′ and 3′ primers, respectively. This *Kpn*I–*Xho*I fragment was then cloned into p2T7-177 digested with *Xho*I and *Kpn*I. eGFP was amplified from pGad8 [34], the 5′ primer was designed to incorporate a *Hin*dIII site followed by a start codon and an in-frame TY epitope that was followed by a *Spe*I site which was immediately upstream of the second codon from eGFP. The 3′ primer also incorporated an in-frame TY epitope (and stop codon) between *Xba*I and *Bam*HI sites. This PCR product was cloned as a *Hin*dIII *Bam*HI fragment into the p2T7-177/pLEW100 hybrid vector cut with *Hin*dIII and *Bam*HI to remove the luciferase gene. Subsequent fluorescent protein genes were cloned into the *Spe*I *Xba*I sites to replace the eGFP. For the amplification of open reading frames, the standard primer design was: forward primer, 5′-AAGCTTCCGCCACCATG followed by the next 15 bases of the ORF. The *Hin*dIII site and the initiation codon are underlined. Reverse primer: 5′-GGATCC AGAACC followed by reverse complement of last 18 bases of the ORF. The *Bam*HI site is underlined, the spacer sequence was replaced with a stop codon if required. All amplified ORFs were cloned and sequenced prior to subcloning into expression vectors.

The derivatives made from the three vectors for the expression of tagged proteins are shown in Fig. 1 and listed in Table 1. All the vectors derived from pLEW100 and pDex377 were designed to accept an open reading frame (ORF) as a *Hin*dIII *Bam*HI fragment and in all these vectors the open reading frames can be transferred from one vector to another without loss of reading frame. The fusion protein has a linker of 5–8 residues between ORF and the tag.

## 2 New vectors for constitutive expression of transgenes from the endogenous gene locus

**Fig. 2:**
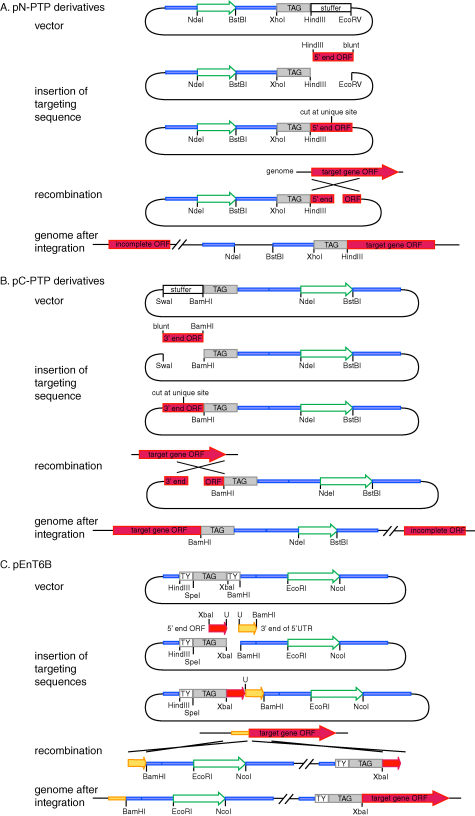
Diagram showing design and use of endogeneous locus tagging vectors. Green arrow, selectable marker gene; red, targeted open reading frame; yellow, 5′ sequences of targeted open reading frame. N-terminal tagging vectors were derived from pN-PTPpuro by digesting with *Bst*BI and *Not*I to remove the tubulin intergenic region, an internal *Hin*dIII site, the RPA 5′UTR and PTP tag which were replaced with a polylinker containing *Bst*BI, *Xho*I, *Hin*dIII, *Eco*RV and *Not*I sites. Next, the β to α tubulin inter-ORF region was cloned into the *Bst*BI and *Xho*I sites. Third, a *Hin*dIII *Eco*RV stuffer fragment was cloned into the *Hin*dIII and *Eco*RV sites and finally a range of tags, as *Xho*I *Hin*dIII fragments were cloned into the *Xho*I and *Hin*dIII sites. C-terminal tagging vectors were derived from pC-PTPneo by digesting with Acc65I and *Eco*RI to remove the targeting sequence and tag and replacing with a polylinker containing Acc65I, *Swa*I, *Sma*I, *Bam*HI and *Eco*RI sites. Next, the neomycin phosphotransferase open reading frame, which contained an internal *Bam*HI site, was removed using *Nde*I and *Bst*BI and replaced with a neomycin phosphotransferase open reading frame without a *Bam*HI site. Third, the range of tags available as *Bam*HI *Bgl*II fragments were cloned into the *Bam*HI site and finally a *Swa*I *Bam*HI stuffer fragment was cloned into the *Swa*I *Bam*HI sites. pEnT5 is a derivative of pGad8 [34] and pDex577. pGad8-tubulin was cut with *Xho*I and *Pvu*II, blunt-ended and self ligated. This vector was then cut with *Xba*I and *Spe*I and self ligated (creating pGad8-tubulin-A). pDex577 was cut with *Nhe*I, blunt-ended and then cut with *Hin*dIII. The fragment containing the TY-tagged eGFP and 3′UTR was cloned into the pGad8-tubulin-A cut with *Sma*I and *Hin*dIII, creating pEnT5. The blasticidin marker gene was amplified by PCR with *Eco*RI and *Nco*I sites incorporated into the 5′ and 3′ primers, respectively. This PCR product was cloned into pEnT5 cut with *Nco*I and *Eco*RI (creating pEnT5-1). The actin 5′ intergenic region was then amplified from genomic DNA incorporating *Sph*I and *Eco*RI sites on the 5′ and 3′ primers, respectively. This digested fragment was cloned into pEnT5-1 cut with *Sph*I and *Eco*RI (pEnT5-2). The actin 3′ intergenic region was amplified from genomic DNA incorporating *Nco*I and *Avr*II sites on the 5′ and 3′ primers, respectively. The digested fragment was cloned into pEnT5-2 cut with *Nhe*I and *Nco*I to create pEnT6B. The puromycin resistance marker gene was amplified by PCR using primers incorporating *Eco*RI and *Nco*I sites in the 5′ and 3′ primers, respectively. This digested fragment was cloned into the pEnT6B cut with *Nco*I and *Eco*RI to create pEnT6P.

We have developed two sets of vectors for tagging genes at the endogenous gene locus. The first set were derived from pN-PTPpuro and pC-PTPneo [19] (kind gifts from Arthur Günzl, University of Connecticut) and are listed in Table 1. In these vectors, part of the targeted ORF is cloned in frame with the tag and then the plasmid is linearised using a unique restriction enzyme site within the targeted ORF [19] (Fig. 2). The N-terminal *in situ* tagging vectors were designed to be digested with *Hin*dIII and *Eco*RV such that an N-terminal portion of the targeted ORF could be cloned after digestion with *Hin*dIII and any restriction enzyme that leaves a blunt end. Once the targeted ORF fragment has been inserted, there is a 10-residue linker (–GGGGSQASAT–) between the end of the tag and the initiation codon of the ORF. The C-terminal *in situ* tagging vectors were designed to be digested with *Swa*I and *Bam*HI and the C-terminal part of the targeted ORF cloned as a blunt end-*Bam*HI fragment. Once the targeted ORF fragment has inserted, there is a 5-residue linker (–GGGSG–) between the targeted protein and tag. The reading frames in these vectors are compatible with the *Hin*dIII and *Bam*HI sites present in the pLEW100 and pDex377 derived vectors above and the same amplified ORF can be used.

The second set of vectors for tagging genes at the endogenous locus were based on the new plasmids pEnT5 and pEnT6. These vectors were designed to be highly modular in nature to facilitate: (i) movement of DNA between plasmids; (ii) use of novel tags and (iii) use of endogenous intergenic sequence for tagged protein regulation. The same vector can be used for either N-terminal (using *Xba*I and *Bam*HI) or C-terminal (between *Hin*dIII and *Spe*I) tagging to generate chimeras tagged with both a fluorescent protein and the TY epitope for use in immunolocalisation [22,23]. The plasmids are designed to be used as replacement rather than insertional vectors, as outlined below, to avoid the generation of unwanted gene fragments. This strategy also removes the need for an endogenous linearization site in the targeting fragment (Fig. 2C). For example, to tag proteins at their N-terminus using pEnT5/6, two fragments are amplified from genomic DNA. The first encompasses 250–500 bases from the 5′ end of the target ORF beginning directly at the start or second codon of the ORF. The six bases necessary to form the consensus *Xba*I (or compatible site *Spe*I, *Avr*II, *Nhe*I) site are added to the 5′ end of the 5′ primer in frame with the target gene. A linearization site of choice is then added to the 5′ end of the 3′ primer for the target gene. The second fragment is amplified from the 3′ end of the 5′ intergenic region for the target ORF, from 250 to 500 bp upstream of the ORF to the start codon. The 5′ primer for this fragment incorporates the same linearization site as the 5′ end of the 3′ ORF primer. A *Bam*HI site (or compatible *Bcl*I, *Bgl*II) is added to the 3′ end of the fragment. The two fragments are digested and simultaneously cloned into the pEnT6 vector cut with *Xba*I *Bam*HI. The vector is linearised at the site between the ORF and UTR fragments prior to transfection into *T. brucei* cells. A similar strategy is adopted for C-terminal tagging using the pEnT5/6 vectors. Following insertion of the two fragments required for tagging, the aldolase 3′ intergenic region can be removed by digestion with *Bam*HI and *Sph*I and replaced with the endogenous 3′ intergenic region in order to preserve the endogenous UTR and hence maintain endogenous levels of mRNA. The new plasmid pEnT5 has been sequenced to completion (4× coverage) and all additional tags/modifications were verified by sequencing. These vectors have been used successfully to generate fusion proteins which localize to a variety of subcellular compartments [24–26]. The sequences of these vectors are available from http://web.mac.com/mc115/iWeb/mclab/home.html and http://users.path.ox.ac.uk/∼kgull/index.html.

## 3 Selection of tags

Four fluorescent proteins were chosen: enhanced green fluorescent protein (eGFP; Clontech), enhanced yellow fluorescent protein (eYFP; Clontech), cerulean fluorescent protein (Cerulean FP) [27] and cherry fluorescent protein (Cherry FP) [28]. Two types of tandem affinity purification (TAP) tags were selected: the first (TAP-tag) contains the immunoglobulin binding domain of protein A and a calmodulin binding peptide separated by a tobacco etch virus (TEV) protease site [16]; the second (TAP-PTP) is similar but the calmodulin binding peptide is replaced with a calcium ion dependent monoclonal antibody epitope [19]. Both have been used successfully in trypanosomes [18,19]. The two TAP tags provide alternative strategies should any purification step prove problematic. The third type of tag was based on monoclonal antibody epitopes [29] with commercially available antibodies: three myc tags [30] or six HA tags or twelve HA tags [31]. The vectors for expressing the HA-tagged proteins also included a TEV protease site [32] between the protein and tag to facilitate release of the protein if the tag was used for purification. Finally a tandem tag was made by combining a Strep-tag (a biotin mimic peptide, see http://www.iba-go.com/prottools/prot_fr01_01.html) and twelve tandem HA epitope tags separated by a TEV protease site. Tandem arrays of epitope tags have two advantages: first a stronger signal as there are multiple copies of the epitope, the second is that the longer tandem arrays allow bivalent binding of the monoclonal antibody to a single tagged protein molecule which has the effect of greatly improving the efficiency of immunoprecipitation. The use of tandem epitope tags is not yet well established in trypanosomes but has been used successfully for detection by Western blotting [33].

## 4 Compatibility with cell lines

pLEW100 and pDex577 derivatives require a cell line expressing both TetR and T7RNAP. Derivatives of pDex377 require a cell line expressing TetR only for regulated transgene expression, but can be used in any cell line for high-level constitutive expression. Vectors for tagging genes at the endogenous locus can be used in any trypanosome cell line providing a suitable selectable marker is available. The endogenous locus tagging vectors described here encompass four different drug resistance markers. Moreover, the selectable marker ORFs are readily exchangable, as *Nde*I *Bst*BI fragments in pN-PTPpuro and pC-PTPneo derivatives and as an *Eco*RI *Nco*I fragment in pEnT6, so it is possible to use the vectors in cell lines with several existing drug resistance markers.

## 5 Expression levels of tagged proteins

**Fig. 3:**
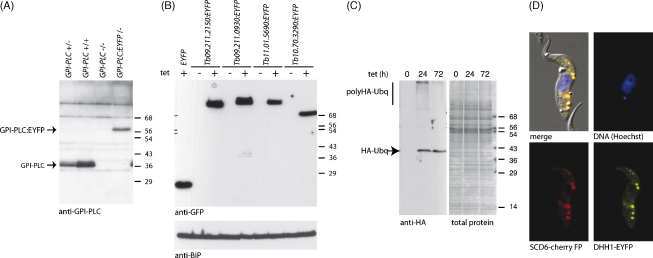
(A) Western blot showing GPI-PLC expression from an endogeneous locus-tagged *GPI-PLC* gene in a heterozygote. (B) Western blot showing the tetracycline-inducible expression of four transgenes from p2216 (Table 1) a pLEW100 derivative adding a C-terminal EYFP tag. (C) Western blot showing the tetracycline-inducible expression of a toxic transgene from pDex377, tandem HA-tagged ubiquitin. (D) Subcellular localisation of DHH1-EYFP and SCD6-CherryFP expressed from two tetracycline-inducible transgenes in the same cell line.

In trypanosomes, most regulation of gene expression is post-transcriptional and the 3′UTR of many mRNAs is believed to be important in determining the half-life and thus the steady state levels of mRNA and encoded protein [34]. The N-terminal tagging of a gene *in situ* leaves the endogenous 3′UTR. Conversely, the C-terminal tagging results in the substitution of the endogenous 3′UTR with that from the RPA1 gene (in the case of pC-PTP derivative) or the aldolase gene (for pEnT6) unless the endogeneous 3′UTR is switched. In either case the protein level is best determined experimentally; for example Fig. 3A shows the expression of GPI-PLC [35,36] with a C-terminal EYFP tag from the endogenous locus and the level is slightly less than the endogenous wild type gene. The second factor that can affect the expression level of the tagged protein is the stability of the fusion protein; to date the vast majority have been stable (Fig. 3B).

Is the expression level from the endogenous gene high enough for visualization of a fluorescent protein fusion? The answer depends on the expression level, the sensitivity of the detecting microscope and whether the protein has a discrete subcellular localisation. The worst-case scenario is when the protein is localized throughout the cell and, from experience, an expression level of ∼2.5 × 10^4^ molecules of a protein located throughout the cytoplasm is required when using a standard laboratory fluorescence microscope.

The level of expression from pLEW100 or pDex377 derivatives is remarkably consistent (Fig. 3B) and roughly equivalent to an abundant cytoplasmic protein, at approximately 1 × 10^5^ molecules per cell in procyclic forms. This estimate of expression level was calibrated by comparison with two eIF4A homologues: using p2280 (Table 1; a pLEW100 derivative adding three myc tags to the C-terminus) expression levels of eIF4AI-myc3 were less than the (2–5) × 10^5^ molecules of endogenous eIF4AI per cell and expression of eIF4AIII-myc3 was greater than the 2 × 10^4^ molecules of endogenous eIF4AIII per cell [33].

## 6 Toxic gene products

The modification of a gene at the endogenous locus results in constitutive expression of a tagged protein; this will not be successful if the tagged protein is lethal to the cell. The tetracycline-regulated EP1 promoter developed in pLEW100 and related vectors has very low levels of expression in the absence of tetracycline [6] (Fig. 3B). The vectors derived from pLEW100 have been used to express dominant lethal mutant proteins that would have killed the cells if the repression had not been effective. As an example, Fig. 3C shows the tetracycline-inducible expression of ubiquitin with a tandem HA tag near the N-terminus. The expression is lethal and the cells have aberrant morphology and cease to proliferate after 3–4 days.

## 7 Functionality of transgenes

Any loss of function in a tagged protein will not always be obvious in a background of untagged protein. An easy way to test whether a tagged gene is functional is to delete one allele and then tag the remaining endogenous allele. A demonstration of the procedure using the *GPI-PLC* gene as an example is shown in Fig. 3A; starting with a wild type cell line, first a heterozygote was produced by targeted gene deletion and then the remaining gene modified by the addition of a C-terminal EYFP tag. The activity of the tagged GPI-PLC was assayed by determining the rate of VSG release from membranes on detergent or hypotonic lysis which were similar to the heterozygote (data not shown).

## 8 Co-expression of more than one transgene

Derivatives of pLEW100, pDex377 and pDex577 can be used to generate cell lines in which there are multiple tetracycline-inducible transgenes. Such cell lines are particularly useful for co-localisation studies. For example, Fig. 3D shows a cell line co-expressing DHH1-EYFP and SCD6-CherryFP and clearly shows co-localisation of the two proteins.

## 9 Conclusions

The development of vectors for functional genomics in trypanosomes is probably equivalent to a teenager; the results do occasionally provide insight but there is still a huge amount to understand and subtlety is lacking. The vectors described here represent a coherent set that will enable more rapid experimental approaches.

## Figures and Tables

**Table 1:** Tags and vectors

Tag	Reference	Vectors for tetracycline-inducible transgenes				Vectors for tagging an endogenous locus	
		pLEW100 C-terminal	pDEX377 C-terminal	pLEW100 N-terminal	pDEX377 N-terminal	C-terminal	N-terminal
Enhanced yellow FP	Clontech	p2216	p2663	p2625	p2628	p2710	p2675
Cerulean FP	[24]	p2619	p2622	p2627	p2630	p2709	p2677
Cherry FP	[25]	p2686	p2664	p2665	p2666	p2705	p2679
TAP	[13]	p2289		p2626	p2629		p2676
TAP PTP	[16]	p2662	p2687			p2706	p2678
3 tandem MYC	[27]	p2280	p2674				
2 tandem TEV:6 tandem HA	[28,29]	p2477	p2623				
2 tandem TEV:12 tandem HA	[28,29]	p2620				p2708	
TAP Strep-tag:2 tandem TEV:12 tandem HA	IBA [28,29]	p2621	p2624			p2707	
Enhanced green FP: TY	Clontech [19,20]	pDex577-G				pEnT5-G pEnT6B-G pEnT6P-G	
Enhanced yellow FP: TY	Clontech [19,20]	pDex577-Y				pEnT5-Y pEnT6B-Y pEnT6P-Y	
Cerulean FP: TY	[19,20,24]	pDex577-C				pEnT5-C pEnT6B-C pEnT6P-C	
